# Incidence and associated factors of cetuximab-induced hypersensitivity infusion reactions in 1392 cancer patients treated in four French areas: a possible association with Lyme disease?

**DOI:** 10.1186/s12885-022-10192-4

**Published:** 2022-11-25

**Authors:** M Dupont, Claire Carlier, C Gower-Rousseau, P Barbier-Lider, D Botsen, M Brasseur, A Burgevin, C Chourbagi, R D’Almeida, V Hautefeuille, M Hentzien, A Lambert, M Lamuraglia, S Lavau-Denes, A Lopez, D Parent, F Slimano, M Brugel, O Bouché

**Affiliations:** 1 Department of Medical Oncology, Godinot Cancer Institute, 1 Rue du Général Koenig, 51100 Reims, France; 2 Department of Gastroenterology and Digestive Oncology, CHU Reims, University of Reims Champagne-Ardenne, Reims, France; 3 Department of Research and Public Health, CHU Reims, Reims, France; 4 Department of Pharmacy, Nancy University Hospital, Vandoeuvre-lès-Nancy, France; 5 Department of Gastroenterology and Digestive Oncology, Nancy University Hospital, Lorraine University, Vandoeuvre-lès-Nancy, France; 6 Department of Pharmacy, Amiens University Hospital, Amiens, France; 7 Department of Gastroenterology and Digestive Oncology, Amiens University Hospital, University of Picardie Jules Verne, Amiens, France; 8 Department of Infectious Diseases and Internal Medicine, CHU Reims, University of Reims Champagne-Ardenne, Reims, France; 9 Department of Medical Oncology, Lorraine Cancer Institute, Vandoeuvre-lès-Nancy, France; 10 Department of Medical Oncology, Amiens University Hospital, University of Picardie Jules-Vernes, Amiens, France; 11 Department of Medical Oncology, Limoges University Hospital, Limoges, France; 12 Department of Pharmacy, Godinot Cancer Institute, Reims, France; 13 Department of Pharmacy, CHU Reims, University of Reims Champagne-Ardenne, Reims, France

**Keywords:** Cetuximab, Infusion reaction, Lyme disease, Hypersensitivity, Risk factors, Alpha-gal, Head, And neck neoplasms

## Abstract

**Background:**

Previous studies have observed an increased incidence of Cetuximab-induced hypersensitivity infusion reactions (CI-IRs) in the southeastern states of the USA. Tick’s bites were suspected of generating cross-reactions between cetuximab and alpha-gal. This study aims was to describe the incidence and associated risk factors of CI-IRs, in the French areas chosen according to their Lyme disease incidence.

**Patients and methods:**

A retrospective chart review was conducted on patients that received cetuximab infusion from January 2010 to June 2019 in 4 French areas with different Lyme disease incidence rates.

**Results:**

Of 1392 patients, 117 (8.4%) experienced a CI-IR, including 68 severe (grade 3 or 4) reactions (4.9%). This CI-IR incidence was significantly higher in the Lyme disease high-risk area than in the other areas (13.2% versus 7.1%, 8.1% and 6.4%; P = 0.016). Sex (P = 0.53), premedication (P = 0.91), primary cancer location (P = 0.46) and chemotherapy regimen type (P = 0.78) had no impact on CI-IR incidence in the overall population. In the head and neck squamous cell carcinoma (HNSCC) patient subgroup, CI-IRs were significantly more frequent in the high-risk area (16.4% versus 6.7%, 7.1% and 7.0%; P = 0.0015).

**Conclusion:**

This study suggests that patients treated in the French area with the highest incidence of Lyme disease are at a higher risk of CI-IRs.

## Highlights

- In a large series of 1392 patients, the 266 patients treated in the French area with the highest incidence of Lyme disease were at a higher risk of cetuximab-induced hypersensitivity infusion reactions compared to those from other areas (16.4% versus 6.7%, 7.1% and 7.0% in medium-, low- and very-low-risk areas; P = 0.0015).
- The risk of cetuximab-induced hypersensitivity infusion reactions in head and neck squamous cell carcinoma patients was higher only in the area with the highest incidence of Lyme disease.
- Age, sex, premedication, primary cancer location and chemotherapy regimen type had no impact on the incidence of cetuximab-induced infusion reactions in the overall population.

## Introduction

Cetuximab is a recombinant human-murine chimeric immunoglobulin G1 monoclonal antibody targeting the human epidermal growth factor receptor (EGFr) by competitively inhibiting the binding of EGF and other ligands on non-tumoral and tumoral cells [1]. Cetuximab has been approved for the treatment of metastatic colorectal cancer in association with anticancer chemotherapy regimen (only for wild type Rat Sarcoma Virus (RAS) status) and advanced head and neck squamous cell carcinoma (HNSCC), in combination with radiotherapy or platinum-based anticancer chemotherapy regimen [1–8].

The most common adverse events include cutaneous eruptions and hypersensitivity infusion reactions [2, 9–11]. Cetuximab-induced hypersensitivity infusion reactions (CI-IRs) occurred in 8.4% of 1373 patients who received cetuximab across clinical trials, mostly on first infusion [1]. Among them, severe CI-IRs were reported in 2.2% with one fatal outcome [1]. An anaphylactic mechanism mediated by immunoglobulin E seems to be a predominant pathway in those hypersensitivity reactions [12–16]. CI-IR incidence could be reduced using combined corticosteroid and histamine H-1 antagonist (H1A) premedication in metastatic colorectal cancer [2, 17–20]. A systematic double premedication is now recommended in Summary Product Characteristics (SPC) of cetuximab. The SPC also specifies an infusion flow rate of 5 and 10 mg/min for first and subsequent courses respectively to prevent CI-IRs [2].

Geography, allergy history, cancer type (HNSCC), ethnic group, premedication, tobacco and alcohol consumption have been reported as potential risk factors for CI-IRs, but these factors remain controversial [21–29].

In southeastern states of the United State of America (USA), an increased incidence of CI-IRs was observed in real life during post-marketing surveillance [21–23, 26, 28, 30–33]. High rates of severe CI-IRs have been reported in North Carolina (14.4%), Tennessee (14%), Missouri (24.6%), Oklahoma (12.4%), Florida (27%) and Arkansas (14.5%) [21, 23, 26, 28, 30, 33]. Hypotheses have been put forward that a history of lone star tick (Amblyomma americanum) bites prior to cetuximab infusion could generate crossed reactions between cetuximab and alpha-gal, an oligosaccharide also observed in cetuximab heavy chain, through IgE [12, 13, 15, 16, 31, 34–39, 40, 41].

The European data related to CI-IR incidence and geographic area as a risk factor are poor [27, 29]. In Europe, the Ixodes ricinus tick is responsible for transmitting the Borellia burgdorferi bacterium causing Lyme disease [42]. This European tick is also associated with IgE responses to alpha-gal and red meat allergy [39, 43–46]. Variability in incidence rates of Lyme disease could be explained by differences in geographical and climate characteristics, in types of exposure and the presence of competent reservoir hosts [47].

The aims of the present CETUXIR study were to describe the incidence of CI-IRs, their management and associated risk factors in four French areas with different Lyme disease incidence rates.

## Patients and methods

### Study design and patients

**Fig. 1:**
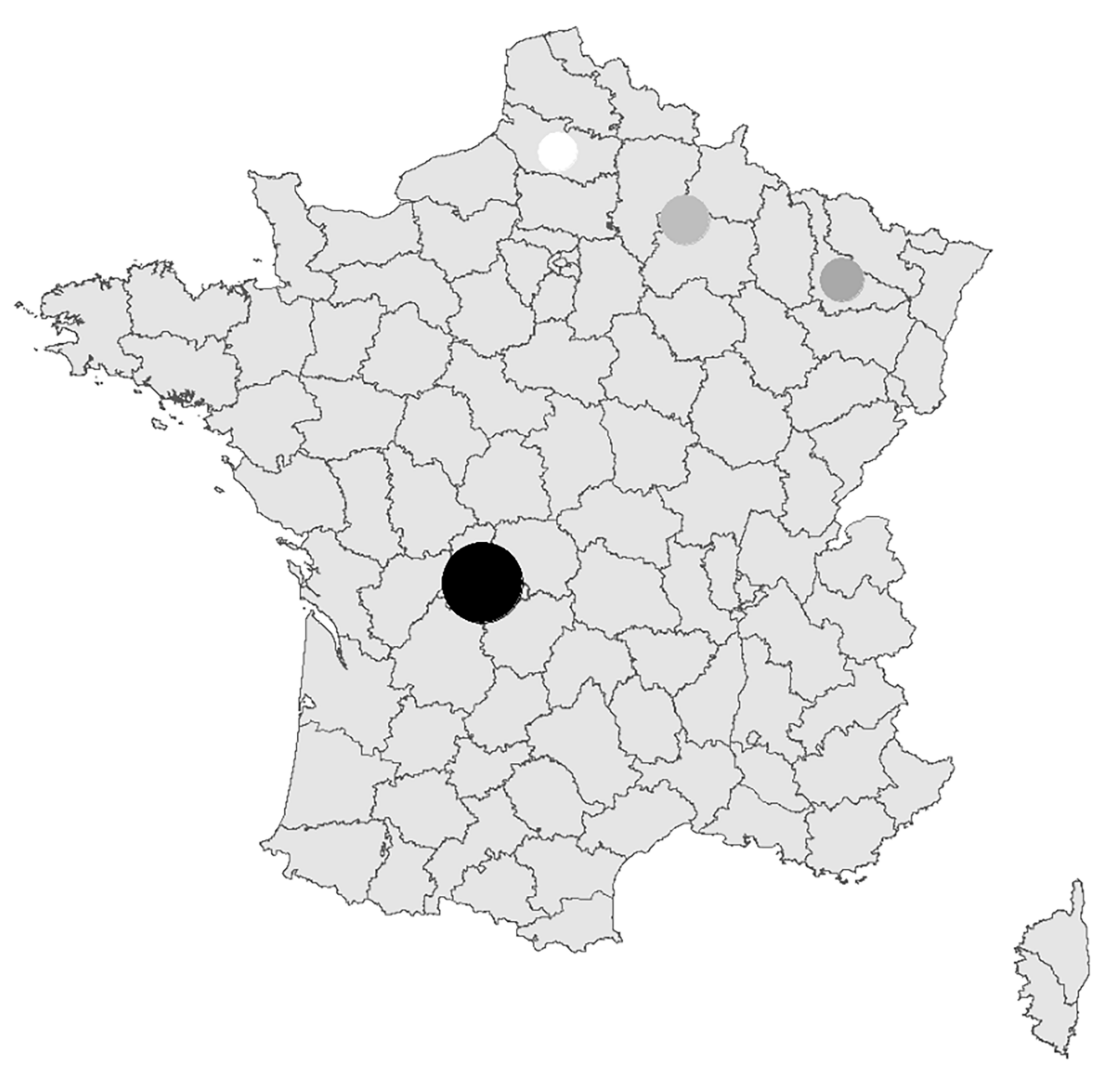
French map with estimated mean annual regional incidence rates of Lyme disease (per 100 000 inhabitants) and incidence of cetuximab-induced hypersensitivity infusion reactions (CI-IRs) in each of the four French areas CI-IRs, cetuximab-induced hypersensitivity infusion reactions; N, number of patients

The CETUXIR study was a retrospective multicentric cohort study of all consecutive cancer patients treated with cetuximab conducted at 6 tertiary centres, in four French areas (defined by administrative regions): Amiens University Hospital, Limoges University Hospital, the University Hospital and Lorraine Cancer Institute in Nancy, the University Hospital and Godinot Cancer Institute in Reims. Areas were chosen and ranked in distinct categories according to their known Lyme disease incidence, using reference work from epidemiological surveillance by the Sentinelles network between 2005 and 2016: high risk (> 150/100,000 inhabitants) for the Limoges area, medium risk (50–100/100,000 inhabitants) for the Nancy area, low risk (20–50/100,000 inhabitants) for the Reims area and very low risk (5–20/100,000 inhabitants) for the Amiens area [47] (Fig. 1).

Inclusion criteria included patients over 18 years old undergoing the administration of a first infusion of cetuximab from 1st January, 2010 to 30th June, 2019. Patients were excluded if they were opposed to the study.

## Data collection

Data from eligible patients were extracted and collected from the computerized order entry (CPOE) software for anticancer chemotherapy (CHIMIO v5.7, Computer Engineering, Paris, France). The following clinical, pathological and therapeutic variables were collected: age, sex, primary cancer location, cetuximab-based regimen type (monotherapy or chemotherapy-based combination, concomitant radiotherapy) and use of premedication with corticosteroids and/or H1A. Lyme disease history of the included patients were not collected. Finally, the occurrence and symptoms of CI-IRs (any grades) on the first infusion of cetuximab and outcome (potential cetuximab rechallenge, desensitization procedure, and switch to panitumumab) were recorded. CI-IRs were graded retrospectively based on National Cancer Institute Common Terminology Criteria for Adverse Events (NCI-CTC-AE) version 5.0 using medical records or via symptom review [48]. Severe CI-IRs were defined as grade 3- and 4- reactions.

### Outcomes

The primary objective was to describe the incidence of CI-IRs in different French areas according to Lyme disease incidence. The secondary objectives were to describe therapeutic management after CI-IRs and to identify CI-IR-associated risk factors.

### Ethical considerations

Patients’ records were anonymized prior to analysis. A database was created in accordance with the reference methodology MR004 of the National Commission of Liberties and Informatics (n°2,206,749, 13/09/2018). A non-opposition form was sent to each living patient included in the study. As per French regulations, no additional ethical committee review was required.

### Statistical analysis

Quantitative data were described with their median and inter-quartile ranges (Q1-Q3) and compared with the non-parametric Kruskall-Wallis test. Qualitative data were described by frequencies and percentages and compared with the Chi-square test or Fisher’s exact test when appropriate. Subgroup analyses of CI-IR risk factors by area were carried out. Multivariate analyses were conditioned to a minimal number of events at the statistician’s discretion. All analyses were performed using SAS version 9.4 (SAS Institute Inc., Cary, NC, USA). Statistical significance was defined as a P-value < 0.05 for all tests.

## Results

### Population characteristics

A total of 1392 consecutive patients were included. The main characteristics of patients treated by cetuximab, overall and per area, are presented in Table 1. Most patients were male (78.4%) and received cetuximab in combination with polychemotherapy (65.5%). Most primary cancer locations were advanced HNSCC (69%) or metastatic colorectal cancer (24%). Almost all patients (97.8%) received double premedication (corticosteroids and H1A). Mean age was 63 + 10 years, but patients were younger in the medium-risk area (61.6 + 10; P = 0.006). Frequencies of HNSCC and colorectal cancer were significantly different in the 4 areas (for HNSCC: 59.8% in the high-risk area, 70.7% in the medium-risk area, 69.5% in the low-risk area and 73.8% in the very-low-risk area; P = 0.0003; and for colorectal cancer: 37.2% in the high-risk area, 24.4% in the medium-risk area, 19.4% in the low-risk area, and 21.8% in the very-low-risk area; P < 0.0001. Double premedication frequency was also significantly different in the 4 areas (99.6% in the high-risk area, 98.9% in the medium-risk area, 94.3% in the low-risk area and 99.2% in the very-low-risk area, P < 0.0001) (Table 1).

**Table 1:** Population characteristics and univariate analysis on the association between population characteristics and 4 areas of treatment

Characteristics		Total *(N = 1392)*	High-risk area^a^ Limoges *(N = 266)*	Medium-risk area^b^ Nancy *(N = 467)*	Low-risk area^c^ Reims *(N = 407)*	Very-low-risk area^d^ Amiens *(N = 252)*	***P***-value	
Age (years), median (inter-quartile)		63.0 (56–70)	64.5 (57–72)	62.0 (55–68)	63.0 (56–71)	63.0 (58–70)	0.0064	
Sex								
	Male, *N* (%)	1091 (78.4)	199 (74.8)	368 (78.8)	329 (80.8)	195 (77.4)	*NS*	
	Female, *N* (%)	301 (21.6)	67 (25.2)	99 (21.2)	78 (19.2)	57 (22.6)	*NS*	
Primary cancer locations								
	HNSCC, *N* (%)	958 (68.8)	159 (59.8)	330 (70.7)	283 (69.5)	186 (73.8)	0,003	
	Colorectal, *N* (%)	347 (24.9)	99 (37.2)	114 (24.4)	79 (19.4)	55 (21.8)	< 10^− 4^	
	CSCC, *N* (%)	65 (4.7)	7 (2.6)	8 (1.7)	44 (10.8)	6 (2.4)	*NS*	
	Cervical, *N* (%)	2 (0.1)	0	0	1 (0.3)	1 (0.4)	*NS*	
	Other, *N* (%)	20 (1.4)	1 (0.4)	15 (3.2)	0	4 (1.6)	*NS*	
Premedications								
	Corticosteroids alone, *N* (%)	19 (1.4)	1 (0.4)	1 (0.2)	15 (3.7)	2 (0.8)	*NS*	
	H1A alone, *N* (%)	11 (0.8)	0	4 (0.9)	7 (1.7)	0	*NS*	
	Corticosteroids + H1A, *N* (%)	1361 (97.8)	265 (99.6)	462 (98.9)	384 (94.6)	250 (99.2)	< 10^− 4^	
Types of chemotherapy								
	Monotherapy, *N* (%)	49 (3.5)	11 (4.1)	5 (1.1)	32 (7.9)	1 (0.4)	*NS*	
	Polychemotherapy, *N* (%)	911 (65.5)	205 (77.1)	335 (71.7)	198 (48.6)	173 (68.6)	*NS*	
	Concomittant radiotherapy, *N* (%)	432 (31)	50 (18.8)	127 (27.2)	177 (43.5)	78 (31)	*NS*	

*N*, number of patients; HNSCC, head and neck squamous-cell carcinoma; CSCC, cutaneous squamous-cell carcinoma; H1A, histamine-1 receptor antagonist; NS, not significant. ^a, b, c, d^ defined according to estimated mean annual regional incidence rates of Lyme disease from Septfons A, et al. Eurosurveillance. 2019;24(11). doi:10.2807/1560-7917.ES.2019.24.11.1800134 [41] : ^a^ > 150 / 100,000 inhabitants; ^b^ 50–100 / 100,000 inhabitants; ^c^ 20–50 / 100,000 inhabitants; ^d^ 5–20 / 100,000 inhabitants

### Incidence of CI-IRs

**Fig. 2:**
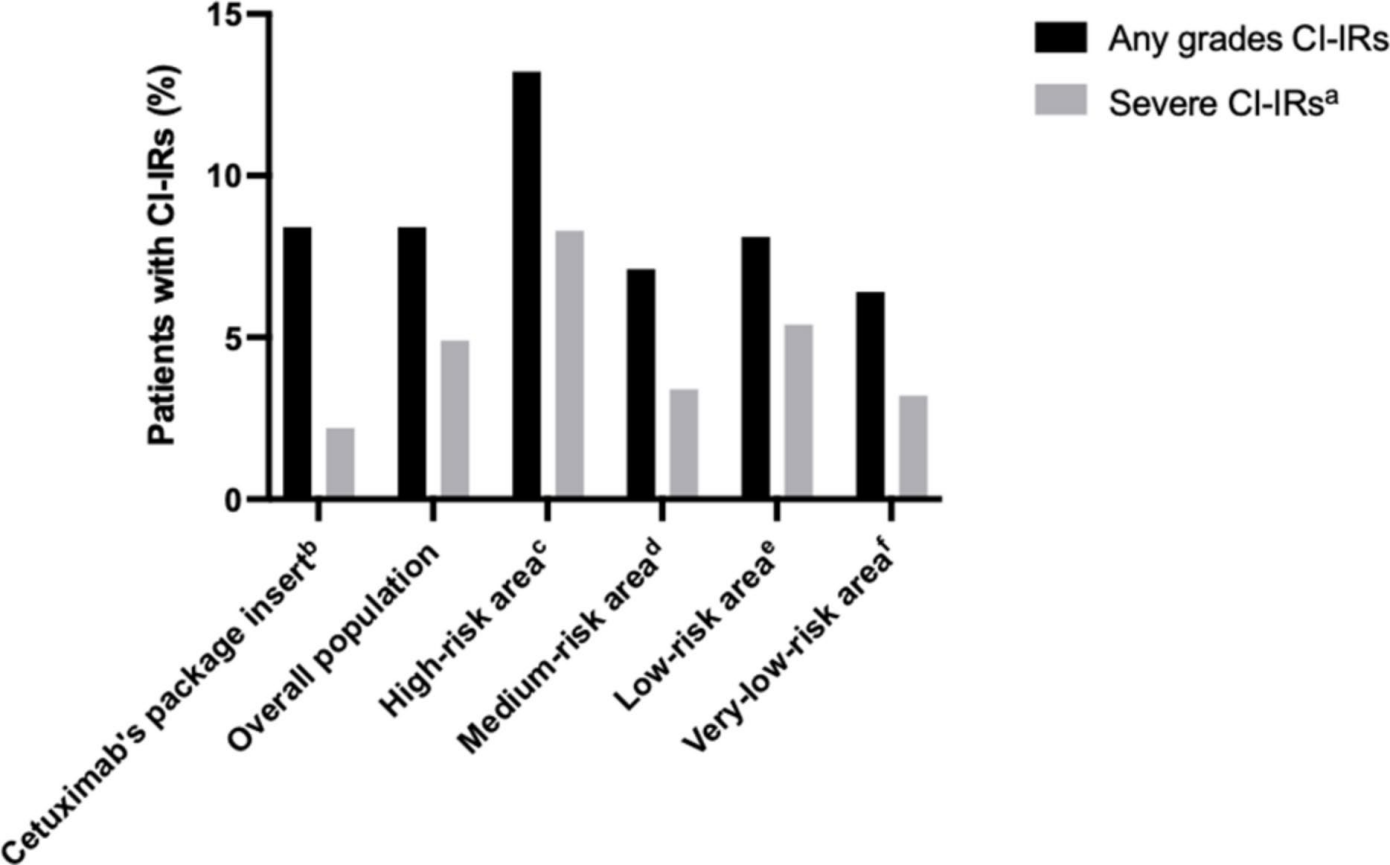
Distribution of any grades and severe grades of cetuximab-induced hypersensitivity infusion reactions (CI-IRs) in each area with different Lyme disease risks (N = 117) CI-IRs, cetuximab-induced hypersensitivity infusion reactions. a defined as grade 3 or grade 4 infusion reactions. b from ImClone Systems Incorporated. U.S. Food and Drug Administration. Erbitux (cetuximab) prescribing information. 2019 [1]. c, d, e, f defined according to estimated mean annual regional incidence rates of Lyme disease from Septfons A, et al. Eurosurveillance. 2019;24(11). doi:10.2807/1560-7917.ES.2019.24.11.1800134 [41] : c > 150 / 100,000 inhabitants; d 50–100 / 100,000 inhabitants; e 20–50 / 100,000 inhabitants; f 5–20 / 100,000 inhabitants

Of the 1392 patients, 117 (8.4%) experienced one CI-IR including 68 (4.8%) severe infusion reactions. No fatal outcome occurred. CI-IR incidences that occurred among patients in each area according to their grade are presented in Fig. 2; Table 2. Incidence of CI-IRs of any grade was significantly higher in the Limoges high-risk area of Lyme disease (13.2% versus 7.1%, 8.1% and 6.4%; P = 0.016). Severe CI-IRs were also more frequent in the high-risk area (8.3% versus 3.4%, 5.4% and 3.2%; P = 0.04) (Fig. 2).

**Table 2:** Incidences and grades of cetuximab-induced hypersensitivity infusion reactions (CI-IRs) in each area with different Lyme disease risks

Areas	Estimated mean annual regional incidence rates of Lyme disease^a^*(per 100 000 inhabitants)*	Incidences of CI-IRs							
		Grade 1, *N* (%)	Grade 2, *N* (%)	Grade 3, *N* (%)	Grade 4, *N* (%)	Grade 5, *N* (%)	Low grade^b^, *N* (%)	Severe grade^c^, *N* (%)	Any grades, *N* (%)
High-risk area Limoges *(N = 266)*	> 150	3 (1.1)	10 (3.8)	14 (5.3)	8 (3.0)	0	13 (4.9)	22 (8.3)	35 (13.2)
Medium-risk area *Nancy (N = 467)*	50–100	7 (1.5)	10 (2.1)	14 (3.0)	2 (0.4)	0	17 (3.6)	16 (3.4)	33 (7.1)
Low-risk area Reims *(N = 407)*	20–50	4 (1.0)	7 (1.7)	14 (3.4)	8 (2.0)	0	11 (2.7)	22 (5.4)	33 (8.1)
Very-low-risk area Amiens *(N = 252)*	5–20	4 (1.6)	4 (1.6)	5 (2.0)	3 (1.2)	0	8 (3.2)	8 (3.2)	16 (6.4)
Overall study population (*N* = 1392)		18 (1.3)	31 (2.2)	47 (3.4)	21 (1.5)	0	49 (3.5)	68 (4.9)	117 (8.4)

CI-IRs, cetuximab induced hypersensitivity infusion reactions; *N*, number of patients

^a^ from Septfons A, et al. Epidemiology of Lyme borreliosis through two surveillance systems: the national Sentinelles GP network and the national hospital discharge database, France, 2005 to 2016. Eurosurveillance. 2019;24(11). doi:10.2807/1560-7917.ES.2019.24.11.1800134 [41]. ^b^ defined as grade 1 or grade 2 infusion reactions. ^c^ defined as grade 3 or grade 4 infusion reactions

### Therapeutic management after CI- IRs

**Fig. 3:**
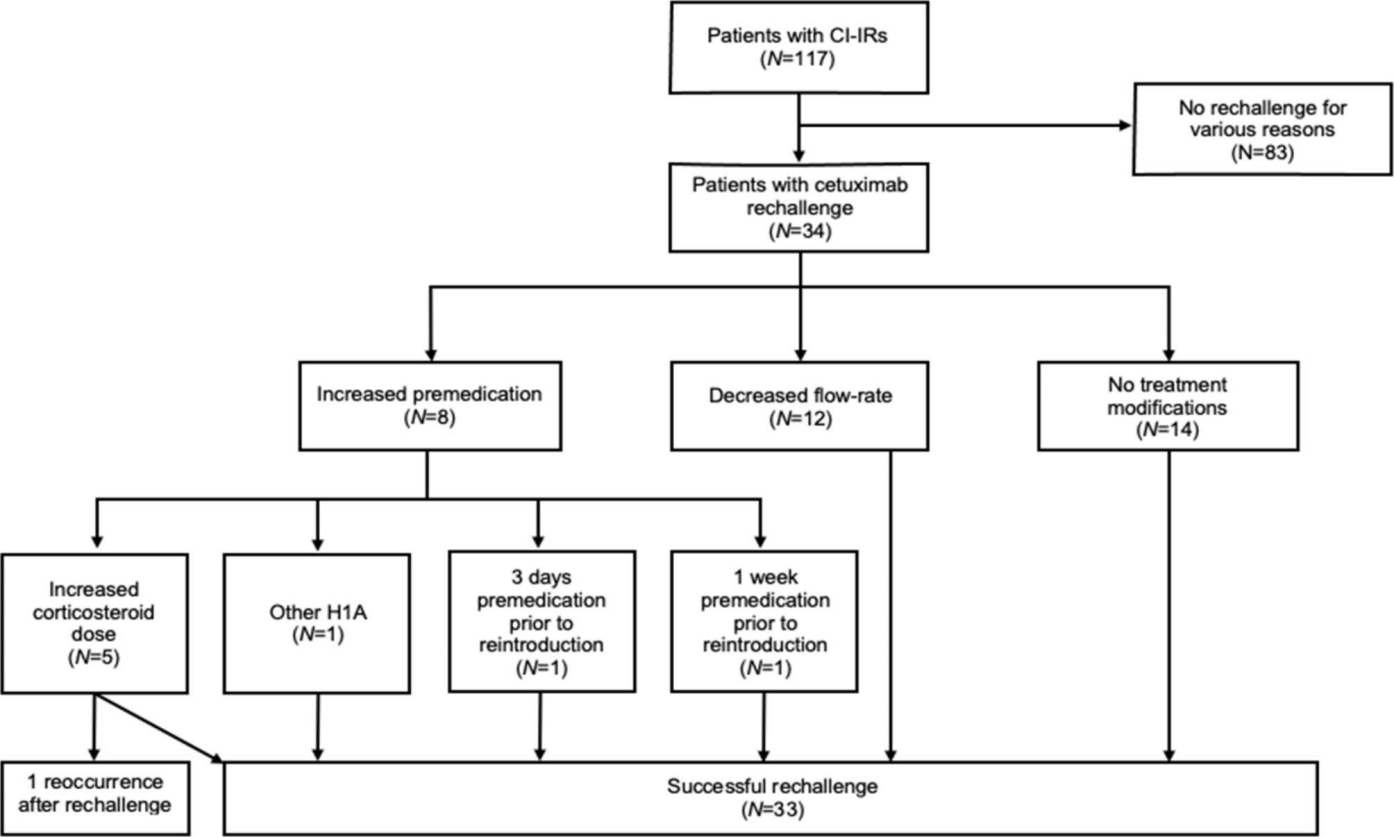
Flow chart of cetuximab rechallenge after cetuximab-induced infusion reaction (CI-IRs). CI-IRs, cetuximab-induced infusion reactions; N, number of patients; H1A, histamine-1 receptor antagonist

The flow chart of cetuximab rechallenge after cetuximab-induced infusion reaction (CI-IRs) is presented in Fig. 3. Thirty-four patients among 117 (29%) who had a CI-IR were rechallenged. Among them, 8 patients were rechallenged with increased premedication and 12 patients received cetuximab with a decreased flow-rate infusion. A total of 14 patients were rechallenged without treatment modification. Only one patient experienced a subsequent reaction despite premedication reinforcement. In this study, no patient was desensitized. Among the 30 patients treated for metastatic colorectal cancer, 13 (43%) were switched to panitumumab without infusion reaction.

### Associated variables with CI-IRs

In the overall population, age, sex, primary cancer location, premedication type and cetuximab-based regimen were not significantly associated with CI-IRs (Table 3).

**Table 3:** Univariate analysis on the risk factors of cetuximab-induced hypersensitivity infusion reactions (CI-IRs)

Characteristics		N	Patients with CI-IRs *(N = 117)*	Patients without CI-IRs *(N = 1275)*	***P***-value	
Age (years), mean ± SD		1392	64.0 ± 9.7	62.9 ± 10.6	0.28	
Sex					0.53	
	Male, *N* (%)	1091	89 (8.2)	1002 (91.8)		
	Female, *N* (%)	301	28 (9.3)	273 (90.7)		
Primary cancer locations					0.91	
	Head and Neck, *N* (%)	958	81 (8.5)	877 (91.5)		
	Colorectal, *N* (%)	347	30 (8.6)	317 (91.3)		
Premedications					0.46	
	Corticosteroids alone, *N* (%)	19	0	19 (100)		
	H1A alone, *N* (%)	11	0	11 (100)		
	Corticosteroids + H1A, *N* (%)	1361	117 (8.6)	1244 (91.4)		
Types of chemotherapy regimen					0.78	
	Monotherapy, *N* (%)	49	4 (8.2)	45 (91.8)		
	Polychemotherapy, *N* (%)	911	80 (8.8)	831 (91.2)		
	Concomittant radiotherapy, *N* (%)	432	33 (7.6)	399 (92.4)		
Lyme disease risk areas^a^					0.016	
	High-risk area^b^, *N* (%)	266	35 (13.2)	231 (86.8)		
	Medium-risk area^c^, *N* (%)	467	33 (7.1)	434 (92.9)		
	Low-risk area^d^, *N* (%)	407	33 (8.1)	374 (91.9)		
	Very-low-risk area^e^, *N* (%)	252	16 (6.4)	236 (93.6)		

CI-IRs, cetuximab induced hypersensitivity infusion reactions; SD, standard deviation; H1A, histamine-1 receptor antagonist

^a^ defined according to estimated mean annual regional incidence rates of Lyme disease from Septfons A, et al. Epidemiology of Lyme borreliosis through two surveillance systems: the national Sentinelles GP network and the national hospital discharge database, France, 2005 to 2016 Eurosurveillance. 2019;24(11). doi:10.2807/1560-7917.ES.2019.24.11.1800134 [41]. : ^b^ > 150 / 100 000 inhabitants; ^c^ 50–100 / 100 000 inhabitants; ^d^ 20–50 / 100 000 inhabitants; ^e^ 5–20 / 100 000 inhabitants

All 117 patients who experienced a CI-IR received the recommended double premedication.

In the HNSCC patient subgroup, CI-IRs were more frequent in the high-risk area of Lyme disease (16.4%) than in the 3 other areas (6.7% in the medium-risk area, 7.1% in the low-risk area and 7.0% in the very-low-risk area; P = 0.0015). There was no significant difference in CI-IR incidence concerning the colorectal patient subgroup in the four areas (8.1% in the high-risk area, 9.7% in the medium-risk area, 11.4% in the low-risk area and 3.7% in the very-low-risk area), nor concerning sex, cetuximab-based regimen and pre-medication subgroups (P not evaluable (futile); data not shown).

## Discussion

The CETUXIR study is the first one to investigate CI-IR distribution in a large cohort of 1392 consecutive patients treated in real life in four different French areas. Our study shows a higher incidence of CI-IRs in the area with the highest risk of Lyme disease in a European country, in line with previous non-European findings.

The present study found an overall incidence of 8.4% of any grades CI-IRs, consistent with previous data provided by the cetuximab SCP [1] (Fig. 2). CI-IR rates were significantly higher in the high-risk area of Lyme disease compared with others (13.2% versus 7.1%, 8.1%, 6.4%; P = 0.016) and compared with the literature [1, 2, 4, 5, 7, 49–51]. Severe CI-IR rates also seemed to be higher in the high-risk area (8.3% versus 3.4%, 5.4% and 3.2%). However, no statistical test was performed for severe CI-IRs due to small samples per group despite the large number of patients in the study. A previous French monocentric study found a 5.4% rate of CI-IRs in 428 patients treated for advanced HNSCC at Gustave Roussy cancer centre in Paris, Île de France (urban low-risk area) [29]. In another French small cohort of 229 patients living in the Normandy very-low-risk area, the CI-IRs rates were 10.5% (any grades) and 4.8% (grades 3–4) respectively [52]. In this study, no clinical variables predicted CI-IR risk but anti-cetuximab IgE was found in 13 of 17 patients (76.5%) when experiencing CI-IRs compared with 17 of 91 control patients (18.7%).

Our results suggest the role of Lyme disease and tick bites in the occurrence of CI-IRs. An increased rate of CI-IRs has also been largely studied in the southeastern states of the USA in which the distribution of the Amblyomma americanum tick (lone star tick) overlaps with the region of both cetuximab sensitivity and red meat allergy [21, 22, 26, 28, 30, 33, 34]. The Ixodes ricinus tick is the main European vector of Lyme disease (a borreliosis) and is also associated with alpha-gal sensitization. In Sweden, Hamsten and al. screened 207 patients with Lyme disease as a confirmed recently tick-bitten population and found 22% to have positive IgE levels to alpha-gal [46]. In a second study, Hamsten and al. demonstrated that the alpha-gal epitope was present in the gastrointestinal tract of Ixodes ricinus [43].

Despite the identification of different individual risk factors for CI-IRs, these associated factors are not consistent in retrospective studies [22, 24, 25, 28, 33]. Among the 374 CI-IRs reported during a 15-year period in The French Pharmacovigilance database, the indication of cetuximab was more likely HNSCC than colorectal cancer (P < 0.001) [27]. In a French monocentric study, combined tobacco and alcohol history (P = 0.009) and prior allergy history (P = 0.003) were associated with CI-IRs in 428 patients treated for HNSCC [29]. The pathophysiological mechanisms of the relation between CI-IR risk and HNSCC with alcohol and tobacco history remain unclear. Tobacco and alcohol exposure could mediate local chronic inflammation and favour an IgE-mediated reaction [29]. In the present study, tobacco-alcohol consumption was not collected and the risk of CI-IRs in HNSCC patients was higher only in the Limoges high-risk area. This result suggests the existence of a still unknown confusion bias between HNSCC and Lyme disease.

The ability of double premedication to reduce the incidence and severity of Ci-IRs was not clear according to current published data [17–19, 21, 22, 24, 28, 52]. Although the administration of H1A before infusion of cetuximab may limit the occurrence of anaphylaxis, the addition of corticosteroids does not seem to be effective. Interestingly, in our study, all the patients who experienced a CI-IR received a double premedication with corticosteroid and H1A as recommended by SPC and the latest international guidelines [1]. Thus, the high incidence of CI-IRs observed in the Limoges high-risk area was not explained by the use of a non-optimal premedication.

Management of CI-IRs is well described in the cetuximab SPC whatever the grade [1]. However, the present study is in line with previous ones that reported heterogeneous therapeutic management after low-grade (grade 1 and 2) infusion reactions [9, 26, 52, 53]. This might result from the difficulty to detect and input subtle symptoms that occur during low-grade CI-IRs. Some studies have found that the practice of cetuximab rechallenges in low-grade CI-IRs was feasible and safe [24, 53]. Indeed, among the 34 patients who were rechallenged in our study, all experienced low-grade infusion reactions and only one re-challenged patient developed a subsequent reaction. However, it is preferable to avoid cetuximab rechallenge in patients who experienced a severe CI-IR. When performed, it should be justified by suboptimal carcinological outcomes, after drug desensitization. In previous studies, several desensitization protocols were developed to safely carry on using cetuximab [54–60]. Despite this fact, no desensitization procedure was performed in our study and definitive discontinuation of cetuximab was decided for all patients that experienced severe CI-IRs. For patients with metastatic colorectal cancer who experienced CI-IRs, switching to panitumumab seems to be a safe therapeutic alternative, especially in cases of severe CI-IRs [61]. Panitumumab is a fully recombinant IgG2 human monoclonal antibody targeting EGFR with restricted approval to metastatic colorectal cancer treatment alone [62]. Clinical trials have shown that, unlike cetuximab, panitumumab is rarely associated with infusion reactions (3–4%) even without premedication [50, 63–66].

This study has several limitations. First, the rates of CI-IRs might be underestimated due to the retrospective collection of data, as some low-grade IRs (grade 1 or 2) might not have been reported in the health record. Moreover, history of tick bites and anti-cetuximab IgE assay, which could have strengthened this association with chronological and biological arguments were not collected. We were, therefore, unable to compare the incidence of anti-bodies directed against Lyme disease based on the geographical area of the patient. Besides, we considered only variables that could be extracted from clinical databases in our analysis. Additional unmeasured confounding factors (tobacco, alcohol, atopy history, surgery, chemotherapy regimen, radiotherapy doses) contributing to CI-IRs may exist. Furthermore, patients may live or have lived in areas where Lyme disease incidences are different from the area where they were treated. An exhaustive data collection of patients’ current and past locations may be needed.

This study suggests that patients previously infected with Lyme disease are at a higher risk of CI-IRs. HNSCC seems to be a predictive risk factor of CI-IRs only in the Lyme disease high-risk area. This result suggests a possible association between HNSCC and Lyme disease. Further studies are needed to investigate these associations. A geographic and ecological study could be performed to investigate the influence of Lyme disease on the appearance of CI-IRs at the scale of groups of individuals. This retrospective study could be carried out prior to analytical epidemiological studies (cohort or case-control studies carried out at the individual level) to establish a strong association between Lyme disease and CI-IRs. An anti-cetuximab IgE assay could be used prior to cetuximab treatment to identify patients at higher risk of CI-IRs, especially in the area with the highest incidence of Lyme disease.

## Data Availability

All data are availlable by asking the corresponding author at claire.carlier@chu-reims.fr.
